# Obsessive Compulsive Disorder During Coronavirus Disease 2019 (COVID-19): 2- and 6-Month Follow-Ups in a Clinical Trial

**DOI:** 10.1093/ijnp/pyab024

**Published:** 2021-05-28

**Authors:** Lior Carmi, Oded Ben-Arush, Leah Fostick, Hagit Cohen, Joseph Zohar

**Affiliations:** 1 The Post-Trauma Center, Chaim Sheba Medical Center, Israel; 2 The Israeli Center for OCD, Modiin, Israel; 3 The Data Science Institution, The Interdisciplinary Center, Herzliya, Israel; 4 Department of Communication Disorders, Ariel University, Israel; 5 Anxiety and Stress Research Unit, Beer-Sheva Mental Health Center, Faculty of Health Sciences, Division of Psychiatry, Ben-Gurion University of the Negev, Israel

**Keywords:** OCD, CBT, COVID-19

## Abstract

**Background:**

Psychiatric patients are perceived to be especially vulnerable during a pandemic, as it increases stress and uncertainty. Several current publications have considered obsessive-compulsive disorder (OCD) patients to be particularly vulnerable during the coronavirus disease 2019 (COVID-19), and clinicians were advised to adjust treatments accordingly. The purpose of this study was to evaluate the 2- and 6-month impacts of COVID-19 on the symptom severity of OCD patients.

**Methods:**

A cohort of OCD patients actively treated with Exposure and Response Prevention (ERP) combined with pharmacological treatment was evaluated as part of their regular psychiatric assessment twice: 113 patients were evaluated at their 2-month follow-up and 90 patients (from that cohort) were evaluated at their 6-month follow up.

**Results:**

Obsessive-compulsive symptom deterioration was not present in 84% of the patients at the 2-month follow-up and 96% of the patients at the 6-month follow-up. The results were also replicated in the OCD subgroup that included patients with contamination (washers) and illness obsessions, who were believed to be particularly vulnerable considering their obsessional content.

**Conclusions:**

OCD patients (including those with obsessions related to contamination and health) who were under active ERP and pharmacological treatment did not experience exacerbated symptoms during COVID-19 at their 2- and 6-month follow-ups.

**Significance Statement**

This is a 6-month follow-up study of obsessive-compulsive disorder (OCD) patients during coronavirus disease 2019 (COVID-19). The results indicated clinical stability among most of the patients, including those with contamination and illness obsessions. Clinical implications are discussed.

## Introduction

In March 2019, coronavirus disease 2019 (COVID-19) was officially recognized as a worldwide pandemic. Accordingly, lockdown and safety measures, including social distancing and strict hygiene regulations, were taken by many countries. The dramatic increase in health-related stress and economic issues, changes in daily routines, and reduced availability of mental health services has led to increasing concern regarding the psychological effects of the lockdown (Ghebreyesus, 2020; Rubin and Wessely, 2020). Accordingly, the vulnerability of psychiatric patients during the COVID-19 outbreak has been addressed in scientific publications (Wang et al., 2021).

Several publications have considered obsessive-compulsive disorder (OCD) patients to be particularly vulnerable during COVID-19 and expected that they would require special care and altered treatment (Fineberg et al., 2020). The assumption was that since OCD patients are characterized by a feeling of uncertainty (Coles et al., 2005; Hellriegel et al., 2017) and the need to avert danger, COVID-19 would enhance their obsessions and compulsions and consequently result in a higher risk of deterioration among these patients (Banerjee, 2020; Kumar and Somani, 2020). This would be specifically true for those patients with contamination (washer) and illness obsessions, as their obsessional content is related to virus infection (Fineberg et al., 2020).

This rationale is mainly rooted in the link between stress and the exacerbation of psychiatric symptomatology (Williams et al., 1981; Brown and Harris, 2012). The general assumption is that psychiatric patients, who are already less functional during normal routines, are expected to function even less under stressful circumstances. However, the findings from studies conducted during earlier times of external and global stressors (e.g., wars) found no symptomatic exacerbation at times when real, external threats to physical health were present (Schlossberg, 1992; Sasson et al., 1999); therefore, they do not support this view. Indeed, several COVID-19 studies have also found limited exacerbation of obsessive-compulsive symptoms in OCD patients (Benatti et al., 2020; Nissen et al., 2020; Sharma et al., 2020), but all are limited to the initial impact of the pandemic and suffer from methodological shortcomings. Hence, although the course of the current pandemic (COVID-19) is not yet fully understood, its initial (2-month) and 6-month impacts on OCD symptomology can now be assessed.

## Methods

The study was approved by the Institutional Review Board committee of Chaim Sheba Medical Center.

### Participants

All OCD patients from the Israeli Center for OCD who had clinical assessments during April to May 2020 and during September 2020 were evaluated in the study. The participants were diagnosed with OCD by a psychiatrist or psychologist and were under ongoing treatment using Cognitive Behavioral Treatment (CBT) and Exposure and Response Prevention (ERP) in the Israeli Center for OCD clinic (see below). The inclusion criteria were as follows: (1) a primary diagnosis of OCD; (2) being treated for OCD for at least 4 months before the beginning of the COVID-19 crisis and still in ongoing treatment or maintenance with the same clinicians (L.C., O.B.-A., and J.Z.); and (3) using stable medication dosages for at least 2 months (please see Table 1 for the demographic characteristics). The patients were clinically evaluated in person (physically or via Zoom) as part of their treatment routine, and the evaluations were conducted by the same treating psychiatrist or psychologists. In order to achieve coherency in the rating, the interviews were conducted with 2 clinicians in the room (J.Z. and either L.C. or O.B.-A.), and the final score was achieved by agreement.

**Table 1.** Demographic Characteristics of Both OCD Patient Groups

		OCD patients’ 2-month follow-up (n = 113)	OCD patients’ 6-month follow up (n = 90)
Gender, male/female, n		56/57	47/43
Age, range, mean		8–18, 14.3 ± 3.2	9–18, 14.8, ± 3.2 (n = 17)
Child and adolescence (n = 25)		19–73, 33.8 ± 10.5	19–73, 35.6, 33.8 ± 9.6 (n = 73)
Adults (n = 88)			
Marital status^a^ (n)		Single (n = 13)	Single (n = 12)
		Married (n = 36)	Married (n = 34)
General employment^b^		68/84 (80%)	51/69 (73%)
Currently on leave due to pandemic		45\84 (53%)	39/69 (56%)
OCD domains	Harm\aggressive	15 (13%)	12 (13%)
	Sexual\religious	16 (14%)	15 (16%)
	Symmetry\ordering	4 (3%)	1(1%)
	Contamination, illness\washing	46 (41%)	36 (39%)
	Checking\repeating	29 (26%)	26 (29%)
OCRD domains	Trichotillomania	6 (5%)	5(5%)
	Body dysmorphic disorder	2 (2%)	2(2%)
	Skin picking	7 (6%)	5 (5%)
	Hoarding	5(4%)	4(4%)
Medications	Escitalopram 20–40 mg	27(24%)	23(25%)
	Fluoxetine 20–40 mg	11(10%)	8(9%)
	Fluoxetine 60–80 mg	12(11%)	7(8%)
	Fluvoxamine 200–500 mg	11(10%)	9(10%)
	Sertraline 150–200 mg	19(16%)	16(18%)
	Sertraline 250–500 mg	33(29%)	27(30%)
	Aripiprazole augmentation (2.5–5 mg)	74(65%)	63(70%)
	Aripiprazole augmentation (7.5–10 mg)	6(5%)	4(4%)

Abbreviations: OCD, obsessive-compulsive disorder; OCRD, obsessive compulsive and related disorders.

^a^Calculated only for patients above the age of 27 (the average age of marriage in Israel).

^b^Calculated only for patients above the age of 21.

The participants are treated at the Israeli Center for OCD. The treatment includes cognitive and behavioural treatment along with intensive ERP treatment (a few times a week) and family intervention. Patients are also enrolled in a WhatsApp group with their psychologist and ERP trainers and are monitored daily on their progress (i.e., close monitoring takes place even after the patients leave the clinic). All patients are prescribed medium to high dosages of serotonin reuptake inhibitors (SRIs); in some cases, there is an augmentation of small dosages (usually 2.5 mg) of the D2 antagonist aripiprazole (Table 1).

### Clinical assessments

The following Global Clinical Impression–Improvement (CGI-I) questionnaires were given.

1.  To what extent does COVID-19 influence your therapeutic course?

- Very much improved
- Much improved
- Minimally improved
- No influence
- Minimally worse
- Much worse
- Very much worse

2.  What is your level of compliance with the health regulations (e.g., wearing a mask, practicing social distancing, washing hands, keeping the quarantine, etc.) compared to your family/friends/relatives?

- Much more relaxed
- More relaxed
- A little more relaxed
- Same as my surroundings
- A little more strict
- More strict
- Much more strict

3.  Does COVID-19 “affect” your obsession content or compulsions?

- Not at all
- Not
- Probably not
- Not sure
- Probably yes
- Yes
- Definitely yes

### Statistical Analysis

The data analysis was performed using the IBM SPSS 25 software. The clinical evaluation questions for OCD were analyzed using a chi-squared test. The difference between the OCD subgroups in the 3 questions was analyzed using a chi-squared test for independence, with the groups (patients in the contamination and illness domains as 1 group and patients with other [i.e., noncontamination and nonillness] obsessions as a second group) as independent variables and the questions (all 3 of them) and responses as dependent variables. In each question, all 7 answers were analyzed and the required *P* value for significance was corrected (*P*c) for the relevant number of comparisons. A *t*-test was employed to evaluate the mean differences between groups.

## Results

At the 2-month follow-up, a total of 113 OCD patients were evaluated (Table 1). Of these, 65 (58%) were in an active phase of the treatment (ERP 2–3 times a week and active family consultation), while 48 (42%) were in a maintenance phase (clinical monitoring every 1 to 6 months). At the 6-month follow-up, from the initial cohort of 113 patients, 11 patients had finished their active phase of treatment and were not available during the data collection time and 12 patients had stopped treatment (7 due to financial issues and the other 5 patients due to other reasons, none related to COVID-19 issues). Thus, the final analysis was conducted on 90 patients (54 in an active phase and 36 in a maintenance phase).

### Two-Month Follow-Up

#### Clinician Assessment

**Figure 1.**
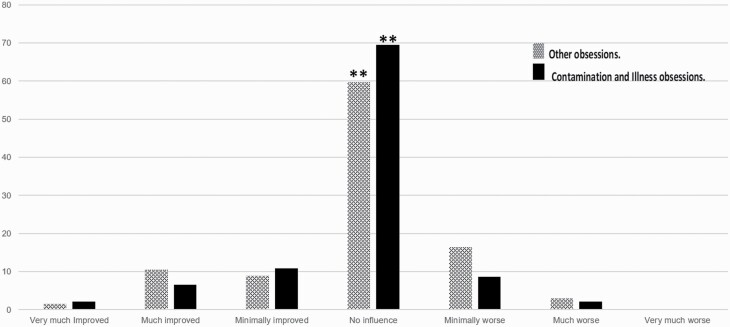
CGI-I scores of OCD patients’ therapeutic course at 2 months following the COVID-19 lockdown. A presentation of the general domains of OCD patients without contamination and illness obsessions (dots), OCD patients with primary contamination and illness obsessions (solid black), and all OCD patients (squares). *P < .001. Abbreviations: CGI-I, Global Clinical Impression–Improvement; COVID-19, coronavirus disease 2019; OCD, obsessive-compulsive disorder.

The number of patients who reported no influence on their therapeutic course (CGI-I) following the COVID-19 lockdown was significantly higher (n = 76/113) than the numbers who provided other answers (very much improved, n = 2/113; much improved, n = 10/113; minimally improved, n = 11/113; minimally worse, n = 11/113; much worse, n = 2/113; and very much worse, n = 1/113; χ2_[6]_ = 266; *P* < .001). Interestingly, the same significant pattern was shown even in those patients suffering primarily from contamination and illness obsessions (n = 46), with no statistically significant difference between the subgroups (χ2_[6]_ = 2.16; *P* = .91). The percentage of each answer for each group is presented in Figure 1.

#### Compliance with Health Regulations

**Figure 2.**
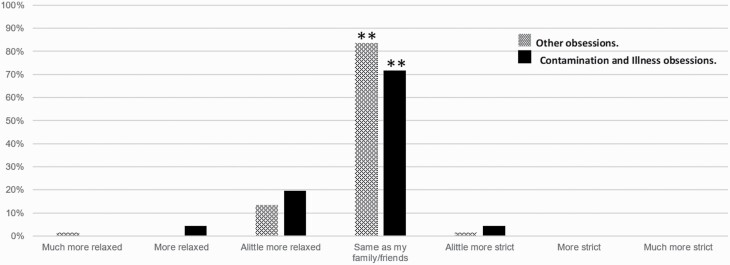
The degree of OCD patients’ compliance with health regulations at 2 months compared to their surroundings following the COVID-19 lockdown. A presentation of the general domains of OCD patients without contamination and hypochondriasis (dots), OCD patients with primary contamination and illness obsessions (solid black), and all OCD patients (squares). *P < .001. Abbreviations: COVID-19, coronavirus disease 2019; OCD, obsessive-compulsive disorder.

Regarding the extent to which OCD patients were compliant with the health regulations, 110 (out of 113) patients reported their actions to be more relaxed or the same as their family and friends, compared to only 3 patients reporting slightly stricter compliance (χ ^2^_[6]_ = 252; *P* < .001). Here, again, a similar pattern was found for those patients with contamination and illness obsessions (Figure 2), with no statistically significant difference between the subgroups (χ ^2^_[6]_ = 2.16; *P* = .91).

#### COVID-19 as an Obsession

**Figure 3.**
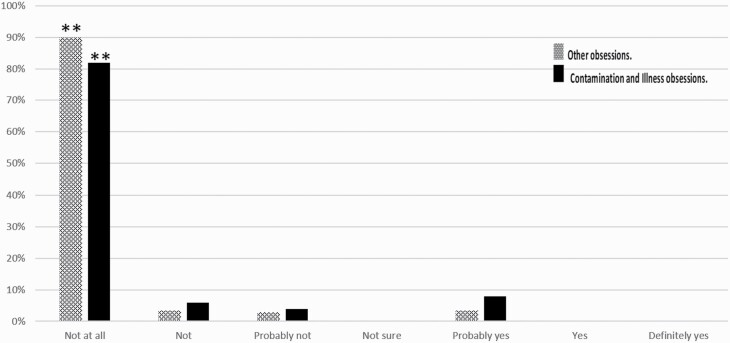
The extent to which COVID-19 became an obsession at 2 months A comparison between general domains of OCD patients without contamination and illness obsessions (dots), OCD patients with primary contamination and illness obsessions (solid black), and all OCD patients (squares). *P < .001. Abbreviations:

The number of OCD patients who reported that COVID-19 did not affect their OCD was significantly higher (not at all, n = 102/113; not, n = 4/113) than the numbers that gave other answers (probably not, n = 3/113; probably yes, n = 4/113; χ ^2^_[3]_ = 256.74; *P* < .001). Here, again, the same significant pattern was seen even in those OCD patients suffering primarily from contamination and illness obsessions (n = 46), with no statistically significant difference between the subgroups (χ ^2^_[3]_ = 7.85; *P*c > .01), taking into account the multiple comparison correction. The percentage of each answer for each group is presented in Figure 3.

In addition, the demographic characteristics and treatment phase did not reach statistical significance in any of the questions (Table 2).

**Table 2.** The Influence of the Demographic Characteristics of OCD Patients at the 2-Month Follow-Up

	Age		Marital status^a^		OCRD		Currently on leave due to the pandemic^b^		Gender		Treatment phase	
	Above 40 (n = 16)	Below 40 (n = 97)	Married (n = 36)	Single (n = 13)	No (n = 93)	Yes (n = 20)	No (n = 43)	Yes (n = 45)	Male (n = 56)	Female (n = 57)	Active (n = 65)	Maintenance (n = 48)
Change in CGI-S, mean	4	3.8	3.9	3.7	3.9	3.7	4	3.7	3.9	3.7	3.9	3.6
	*P* = .87		*P* = .91		*P* = .92		*P* = .8		*P* = .95		*P* = .78	
Health regulations	3.8	3.7	3.9	3.7	3.8	3.8	3.8	3.8	3.8	3.8	3.8	3.7
	*P* = .89		*P* = .92		*P* = .93		*P* = .98		*P* = .9		*P* = .83	
Obsessional thinking	1.2	1.2	1.1	1.3	1.2	1.2	1.2	1.2	1.3	1.2	1.1	1.3
	*P* = .91		*P* = .94		*P* = .9		*P* = .89		*P* = .95		*P* = .91	

Abbreviations: CGI-S, clinical global impression – severity; OCD, obsessive-compulsive disorder; OCRD, obsessive compulsive and related disorders.

^a^Only above the age of 27 (the average age of marriage in Israel).

^b^Only above the age of 21.

### Six-Month Follow-Up

#### Clinician Assessment

At 6 months, the number of patients who reported no influence of the COVID-19 lockdown on their therapeutic course (CGI-I) was significantly higher (no change; n = 65/90; 72.2%) compared to those selecting other answers (very much improved, n = 12/90 [13.3%]; much improved, n = 5/90 [5.5%]; minimally improved, n = 4/90 [4.5%]; minimally worse, n = 1/90 [1.1%]; much worse, n = 2/90 [2.2%]; and very much worse, n = 1/90 [1.1%]; χ2_[6]_ = 286; *P* < .001). Furthermore, the same significant pattern was shown even in those patients suffering primarily from contamination and illness obsessions (n = 36), with no statistically significant difference between the subgroups (χ2_[6]_ = 1.92; *P* = .84).

#### Compliance with Health Regulations

At 6 months, the vast majority of the patients reported that their level of compliance with health regulation was slightly more relaxed or the same as their family and friends (n = 87/90; 96.6%), compared to only 6 patients reporting slightly stricter compliance (n = 3/90; 3.4%; χ ^2^_[6]_ = 261; *P* < .001). Here, again, patients with contamination and illness obsessions did not differ from the general findings, with no statistically significant difference between the subgroups (χ ^2^_[6]_ = 2.22; *P* = .93)

#### COVID-19 as an Obsession

At 6 months, the number of OCD patients that reported that COVID-19 did not affect their OCD was significantly higher (not at all, n = 75/90 [83.3%]; and no, n = 8/90 [8.8%]) than the numbers providing other answers (probably not, n = 3/90 [3.3%]; probably yes, n = 2/90 [2.2%]; and yes, n = 2/90 [2.2%]; χ ^2^_[3]_ = 242; *P* < .001). Here, again, the same significant pattern was seen even in those OCD patients suffering primarily from contamination and illness obsessions (n = 36), with no statistically significant difference between the subgroups (χ ^2^_[3]_ = 5.67; *P*c > .01), taking into account the multiple comparison correction.

No statistically significant difference was found for any of the questions between the 2- and 6-month follow-ups or any of the demographic characteristics.

## Discussion

The present study is, to the best of our knowledge, the first to include longitudinal follow-ups on obsessive-compulsive symptoms in OCD patients at 2 and 6 months from the first pandemic lockdown. We found that 84% of the patients at the 2-month follow-up and 96% of the patients at the 6-month follow-up showed no OCD exacerbation. This finding was not influenced by age, gender, occupational status, or obsessive compulsive and related disorders (OCRD) comorbidities. This pattern was also found in OCD patients who would be intuitively considered to be influenced by the viral pandemic: that is, those with primarily contamination (washer) and illness obsessions.

Our findings are in line those of with previous studies conducted at the 2-month follow-up under the same methodology (i.e., in-person assessment with a clinician). The first (Davide et al., 2020; n = 30) investigated OCD exacerbation in children and adolescents and reported deterioration in 13% of the patients. The second (Matsunaga et al., 2020; n = 60) reported exacerbation in 10% of the cohort. Other studies examining the initial impact of the COVID-19 pandemic on OCD found deterioration in some patients, ranging from 6% (Chakraborty and Karmakar, 2020; Sharma et al., 2020) to 36% (Benatti et al., 2020; Nissen et al., 2020) and 54% (Tanir et al., 2020). However, in all of these studies, patients were evaluated mainly through the telephone or an internet survey (Benatti et al., 2020; Chakraborty and Karmakar, 2020; Nissen et al., 2020; Sharma et al., 2020; Tanir et al., 2020), which exposed the results to a subjective perspective (self-rating vs. clinician rating) and to a biased cohort (only those who agreed to conduct the follow-up or answered the online message).

Our results are also in line with those of prior reports which found that during external and real threats to physical health, psychiatric patients managed to function well. These findings were found for schizophrenic patients who managed to overcome their internal auditory and visual hallucinations while facing real external threats (during the Gulf War in Israel) and functioned as requested and in an organized manner (Melamed et al., 1996). This was also the case for patients with panic disorder, who managed to overcome their suffocation pathology in order to wear gas masks and get into crowded, locked shelters during the Gulf War without developing panic attacks (Sasson et al., 1999). Thus, an optional explanation for our results (and the former results) might be the difference between personal and nonpersonal stressors. This suggests that personal or psychological stress (Cohen et al., 2007; e.g., loss of a job, divorce, etc.) might be experienced with a sense of responsibility and self-blame compared to events in which external and no personal stressor emerges (e.g., rocket attacks, earthquakes, tsunamis, COVID-19, etc.). Thus, while personal stressors tap the pathology and exacerbate the symptoms, nonpersonal stress pushes the internal pathology to the back seat and results in adaptive, lifesaving mechanisms.

Another explanation might be the “pulling together” effect (Joiner et al., 2006). According to this theory, at times of panic in the community, (national/global) threats, and traumatic events, people tend to “pull together”: that is, the feeling of cohesiveness and belongingness increases. Accordingly, this cohesiveness acts as a protective mechanism, and personal stressors at these times tend to be secondary or less dominant.

An alternative explanation for our results might be the contribution of the inherent avoidance element of the quarantine (e.g., less driving, not touching public handles, not using public transportation, etc.), which might have contributed to some extent to the stability of the patients. However, in our clinic, the treatment and exposure continued throughout this time (with necessary adjustments), which also balanced these “legitimate” avoidance behaviours to some extent.

Several limitations need to be mentioned. The first limitation regards the specificity of the study. Our results are limited only to patients who are currently in treatment (whether in the active or maintenance phase). Furthermore, the patients in this study were treated in a private clinic. Those in the acute phase of treatment experienced intensive ERP treatment (up to a few times a week) and close interaction and monitoring of patients throughout the day (via WhatsApp groups). Those in the maintenance phase experienced less intensive treatment (e.g., meetings every couple of weeks). In addition, the treatment was continued (with minor adaptations) during the lockdown. Finally, many of the patients in the Center for OCD work, and all of them were prescribed medium to high dosages of an SRI (some with treatment augmented by a D2 partial agonist). In this regard, our results are limited only to patients who have already participated in intensive treatment for a few months or have been in the maintenance phase of this treatment. Notably, the same pattern of results was maintained for the acute and maintenance phases; accordingly, the generalizability of those results is limited only to patients who are in treatment.

The second limitation regards the absence of a “gold standard” measure (i.e., Yale–Brown Obsessive Compulsive Scale [Y-BOCS]) or validated measures for relapse and remission. However, the fact that the evaluations were made by the authors (L.C., O.B.-A., and J.Z.), who are very familiar with the patients (all were treated by them for more than half a year), might compensate for the absence of these measurements to some extent.

In conclusion, our findings imply that CBT and SRI treatment should be continued, including exposures (considering the health regulations and requirements), at least for patients who are under active treatment. Further studies examining the more chronic effects of COVID-19 and further exploring the different effects of the pandemic on treated and untreated OCD patients (and those with other disorders) are warranted.
